# Novel Replication-Competent Circular DNA Molecules from Healthy Cattle Serum and Milk and Multiple Sclerosis-Affected Human Brain Tissue

**DOI:** 10.1128/genomeA.00849-14

**Published:** 2014-08-28

**Authors:** Corinna Whitley, Karin Gunst, Hermann Müller, Mathis Funk, Harald zur Hausen, Ethel-Michele de Villiers

**Affiliations:** a Division for the Characterization of Tumorviruses, Deutsches Krebsforschungszentrum, Heidelberg, Germany; b Institute for Virology, Faculty of Veterinary Medicine, University of Leipzig, Leipzig, Germany

## Abstract

Epidemiological data point to the involvement of a cow milk factor in the etiology of multiple sclerosis (MS). Eleven circular DNA molecules closely related to transmissible spongiform encephalopathy (TSE)-associated isolate Sphinx 1.76 were isolated from healthy cattle serum, cow milk, and serum and brain tissue from MS patients.

## GENOME ANNOUNCEMENT

The etiology of multiple sclerosis (MS) has not been resolved. Epidemiological data point to the involvement of a cow milk factor (E.-M. de Villiers and H. zur Hausen, unpublished data). We analyzed 120 serum samples from healthy cattle, 4 samples from commercially available milk, as well as serum and brain tissue samples from MS patients, for the presence of yet unknown factors that might play a role in MS etiology.

DNA was extracted by the phenol-chloroform method from milk and the postmortem brain tissue of MS patients. DNA was extracted from all serum samples using the High Pure viral nucleic acid kit (Roche). Pools of 5 bovine serum samples were subjected to density gradient ultracentrifugation, rolling circle amplification (RCA) with random primers on DNA from protein-associated fractions, restriction digestion, cloning, and sequencing of the resulting fragments (1). Three clones shared nucleotide similarity to the transmissible spongiform encephalopathy (TSE)-associated circular DNA isolate Sphinx 1.76 sequence (1,758 bp; accession no. HQ444404) (2). HCBI3.108 (HCBI, healthy cattle blood isolate) (1,086 bp), HCBI4.296 (2,958 bp), and HCBI5.173 (1,723 bp) share 78%, 66%, and 66% nucleotide similarity, respectively, to Sphinx 1.76. As we failed to obtain full-length circular molecules with specific primers, 2 abutting primer pairs were designed on Sphinx 1.76 for PCR on all human and bovine samples: forward, 5′-GGATTAATGCCAATGATCC-3′ (nucleotides [nt] 721 to 739), and reverse, 5′-CGAGAGAAACAGGCAAAG-3′ (nt 703 to 720); and forward, 5′-GAGGACGAATTAATATTACAAGTC-3′ (nt 868 to 891), and reverse, 5′-TTACCAAGAAAAGCGAGAAC-3′ (nt 848 to 867). The resulting isolates sharing nucleotide similarity to Sphinx 1.76 (ranging from 79% to 98%) were the following: two isolates from cattle serum, HCBI6.252 (2,522 bp) and HCBI6.159 (1,591 bp); 4 isolates from milk, CMI1.252 (CMI, cow milk isolate) (2,523 bp), CMI2.214 (2,148 bp), CMI3.168 (1,687 bp), and CMI4.158 (1,583 bp); and 2 isolates from human MS brain tissue, MSBI1.176 (MSBI, multiple sclerosis brain isolate) (1,766 bp) and MSBI2.176 (1,766 bp). MSBI1.176 shares 98% nucleotide similarity to the sequence of Sphinx 1.76. MSBI1.176 is not a laboratory contaminant of Sphinx 1.76 (we do not have the latter available), as the pattern of differences between these 2 sequences indicate sequencing error as being highly unlikely. MSBI1.176 and MSBI2.176 were isolated from one brain sample. HCBI6.252 and HCBI6.159 are identical in the overlapping sequence (deletion of nt 1129 to 2060 in HCBI6.252). The large open reading frames (ORFs) of all isolates encode replication protein (ProtSweep [3]) sharing high similarity between them, except for HCBI4.296 and HCBI5.173, with Rep proteins sharing only 57% and 49% amino acid similarity to the Sphinx 1.76 Rep. HCBI4.296 encodes a second protein (225 amino acids [aa]) with similarity to mobilization proteins. Another common feature is the presence of iteron-like tandem repeats (3 × 22 nt plus 17/18 nt of the repeat in each isolate). The alignment of this repeat region indicates a variation in the core of single nucleotides. These iteron-like repeats may constitute the binding sites for Rep proteins (4, 5).

### Nucleotide sequence accession numbers.

The complete sequences of 11 isolates have been deposited in the EMBL Databank under accession numbers LK931487 (CMI1.252), LK931488 (CMI2.214), LK931489 (CMI3.168), LK931490 (CMI4.158), LK931491 (MSBI1.176), LK931492 (MSBI2.176), LK931495 (HCBI3.108), LK931496 (HCBI4.296), LK931497 (HCBI5.173), LK931493 (HCBI6.252), and LK931494 (HCBI6.159).
